# Phenotypic and genotypic characterization of extended-spectrum beta-lactamase-producing Enterobacteriaceae in a Moroccan hospital

**DOI:** 10.1099/acmi.0.000822.v3

**Published:** 2024-07-17

**Authors:** Yassine Eddair, Elmehdi Belouad, Elmostafa Benaissa, Tilila Abassor, Fatna Bsaibiss, Adil Maleb, Mostafa Elouennass

**Affiliations:** 1 Laboratory of Bacteriology, Mohammed V Military Teaching Hospital, Faculty of Medicine and Pharmacy, Mohammed V University, Rabat, Morocco; 2 Research Team of Epidemiology and Bacterial Resistance, Faculty of Medicine and Pharmacy, Mohammed V University, Rabat, Morocco; 3 Laboratory of Microbiology, Mohammed VI University Hospital, Faculty of Medicine and Pharmacy, University Mohammed the First, Oujda, Morocco

**Keywords:** antimicrobial resistance, ESBL, multi-drug resistant bacteria, phenotypic and genotypic characterization, prevalence

## Abstract

Extended-spectrum beta-lactamase-producing *Enterobacteriaceae* (ESBL-E) is a major public health problem in hospitals and in the community. The objective of this work was to describe the epidemiology of ESBL-E*,* to study their resistance profile and to determine the genes encoding the ESBL phenotype. This is a retrospective study conducted in the bacteriology laboratory of the Mohamed V Military Training Hospital in Rabat, and covering all isolates of *Enterobacteriaceae* from 1 January 2018 to 31 December 2020. The molecular study of ESBL genes involved a representative sample of all ESBL isolates. The overall prevalence of ESBLs in isolated *Enterobacteriaceae* (1402/10268) is 13.65 %. The urinary tract was the main site of isolation of ESBL (61 %). The bacterial species most concerned are *Escherichia coli* (41.9 %), *Klebsiella pneumoniae* (42.2 %) and *Enterobacter cloacae* (11.9 %). The study of antibiotic susceptibility showed a resistant profile marked mainly by 100 % resistance to first generation cephalosporins (1GC) and third generation cephalosporins (3GC), 55 % to piperacillin-tazobactam, 16 % to imipenem, and 87 % to fluoroquinolones. Molecular typing of ESBL strains showed a prevalence of CTX-M (95 %), SHV (50 %) and TEM (56 %). The CTX-M-1 and the CTX-M-9 groups were the most common (96.19 % and 7.62 % respectively), and CTX-M15 was found in 78.10 % of CTX-M-1 ESBL positive isolates. Most strains had more than two coexisting resistance genes. The prevalence rate of ESBL-E is critical, and preventive action at different levels (prescriber, biologist, hospital, patient, etc.) are necessary in order to limit their spread and to manage a better therapeutic strategy.

## Data Summary

No data were reused or generated for this study.

## Note

The present work was the subject of a doctoral thesis in pharmacy by Dr Yassine Eddair.

## Background

Antibiotic resistance in pathogens has become a global problem with serious consequences for the treatment of infectious diseases. The increased use/misuse of antibiotics in human medicine, agriculture and even in veterinary medicine is a major contributor to the phenomenon [1].

*Enterobacteriaceae* is a large group of Gram-negative bacteria widely found in nature and in the human digestive tract. They are generally represented by *Klebsiella pneumoniae, Escherichia coli*, and *Enterobacter cloacae* which are responsible for many infections (urinary, pulmonary, septicemia …) of varying severity. In hospitals, *Enterobacteriaceae* is responsible for more than 30 % of the morbidity and mortality associated with bacterial infections [2].

A high rate of resistance to third generation cephalosporins (3GC) among *Enterobacteriaceae* isolates has been previously reported worldwide [3]. Resistance to β-lactam antimicrobials in *Enterobacteriaceae* has been largely due to the presence of β-lactamase enzymes; an important resistance mechanism that impedes antimicrobial treatment of infections [4]. Extended-spectrum beta-lactamases (ESBLs) are one of the most frequent and widely spread enzyme families that remains a global concern [5]. Their increasing frequency, as well as their continuous evolution, is directly related to the selection through the use of different β-lactams [6].

The majority of ESBLs belong to Ambler’s class A and include SHV or TEM types and CTX-M types [6]. During the 1990s, TEM and SHV ESBL types primarily caused worldwide nosocomial epidemics, but since 2000, the prevalence of CTX-M increased rapidly and is presently the most common global ESBL among enteric bacteria [6].

Currently, CTX-M type β-lactamases include more than 220 different enzymes grouped into five subfamilies based on their amino acid identities: CTX-M-1, CTX-M-2, CTX-M-8, CTX-M-9, and CTX-M-25 [7]. Enzymes that originate from the CTXM-1 and CTX-M-9 subfamilies are widely distributed and commonly reported [6]. Some CTX-M are specific to some countries (such as CTX-M-9 and CTX-M-14 in Spain, CTX-M-1 in Italy, or CTX-M-2 in South America, and Japan) [8] while CTX-M-15 is dominant in most regions worldwide as its emergence and rapid worldwide dissemination has been reported [9]. At the Mediterranean level, CTX-M-15 is today the most frequent of CTX-M type ESBL, involved in community acquired infections as well as in nosocomial infection [10,11], and is responsible for various outbreaks [12]. The CTX-M-15 remains the ESBL gene most identified in Morocco [13,14].

Therefore, the main objectives of our study are to determine the prevalence of extended spectrum beta-lactamases producing *Enterobacteriaceae*, to establish their resistance profile to different families of antibiotics and to determine the different genotypic profiles coding for extended spectrum beta-lactamases.

## Methods

### Patients and bacterial isolates

We conducted a retrospective study spread over a period of 3 years from 1 January 2018 to 31 December 2020, and focused on the totality of *Enterobacteriaceae* isolates from all patients (inpatients and outpatients) whatever their sampling site or originating department. Diagnostic samples were included.

Our study was carried out in the bacteriology laboratory of the Military Hospital of Instruction Mohammed V, a university hospital located in Rabat, Kingdom of Morocco.

Identification of bacterial isolates was based on cultural, morphological and biochemical characteristics. Biochemical identification was performed using ready-to-use API20E strips (bio-Mérieux SA, Marcy-l'Étoile / France).

Duplicates were excluded.

### Susceptibility testing

Antibiotic susceptibility was performed using the Mueller Hinton agar diffusion method using OXOID antibiotic discs and interpreted according to the EUCAST / CA-SFM 2020 recommendations. Quality control of the antibiotic susceptibility test was performed with the *E. coli* strain ATCC 25922.

The antibiotics used are as follows: amikacin, amoxicillin+clavulanic acid, ampicillin, first generation cephalosporin, cefepim, third generation cephalosporin, cefoxitin, fluoroqionolone, ertapenem, fosfomycin, gentamicin, imipenem, mecillinam, piperacillin+tazobactam, tobramycin, trimethoprim+sulfamethoxazole.

### Phenotypic screening for ESBL

The detection of extended-spectrum-β-lactamases (ESBLs) was performed by a phenotypic method based on synergy detection between the amoxicillin-clavulanic acid disc and three cephalosporin discs: cefotaxime, ceftazidime and cefepime.

A sample of third generation cephalosporins-resistant ESBL strains of *Enterobacteriaceae* was studied to search for resistance genes.

### PCR amplification for detection of β-lactamase genes

A random representative sample of 110 phenotypically confirmed ESBL-positive *Enterobacteriaceae* underwent molecular PCR screening for TEM, SHV and CTX-M resistance genes. The selection of isolates was done purely by chance, without any bias or predetermined criteria in such a way that every member of the population has an equal chance of being included, and the sample accurately reflects the characteristics of the whole population.

The PCR reactions were carried out in two steps: a monoplex for the detection of CTX-M gene and a multiplex for the detection of TEM and SHV genes using specific primers (Table 1).

**Table 1.** Nucleotide sequences of PCR primers used to amplify ESBL genes

Gene	Primers	Sequence	Amplicon size (bp)	References
***bla*CTX-M**	CTX-M-F	5'- CGC TTT GCG ATG TGC AG - 3'	551	[43]
	CTX-M-R	5'- ACC GCG ATA TCG TTG GT - 3'		
***bla*TEM**	TEM-F	5′- CAT TTC CGT GTC GCC CTT ATT C - 3′	800	[9]
	TEM-R	5′- CGT TCA TCC ATA GTT GCC TGA C - 3′		
***bla*SHV**	SHV-F	5′- AGC CGC TTG AGC AAA TTA AAC - 3′	713	[9]
	SHV-R	5′- ATC CCG CAG ATA AAT CAC CAC - 3′		

The thermal protocol used is as follows: an initial denaturation at 95 °C for 1 min; followed by 35 cycles of 95 °C for 15 s and 72 °C for 10 s; with a final extension at 72 °C for 10 min. During the 35 cycles, the hybridization temperatures used were 52 °C for the CTX-M monoplex and 58 °C for the SHV TEM multiplex and the hybridization lasted 15 s per cycle.

### Multiplex PCR for CTX-M phylogrouping

CTX-M-positive isolates were further analysed for CTX-M phylogroups (CTX-M-1; -2; -8; -9; -25 and -15) by two multiplex PCR (multiplex one screening for CTX-M-1; -2; -9, and multiplex two for CTX-M-8; -25; -15). Primer pairs and predicted amplicon sizes were summarized in Table 2. The thermal protocol used was: an initial denaturation at 95 °C for 1 min; followed by 35 cycles of 95 °C for 15 s; 54 °C for 15 s and 72 °C for 10 s; with a final extension at 72 °C for 10 min.

**Table 2.** Nucleotide sequences of PCR primers used to amplify CTX-M phylogroups

Gene	Primers	Sequence	Amplicon size (bp)	References
***bla*CTX-M1**	CTX-M1 F	5′-AAA AAT CAC TGC GCC AGT TC-3′	415	[25,44]
	CTX-M1 R	5′-AGC TTA TTC ATC GCC ACG TT-3′		
***bla*CTX-M2**	CTX-M2 F	5′-CGA CGC TAC CCC TGC TAT T-3′	552	[25,44]
	CTX-M2 R	5′-CCA GCG TCA GAT TTT TCA GG-3′		
***bla*CTX-M8**	CTX-M8 F	5′-TCG CGT TAA GCG GAT GAT GC-3′	688	[25,44]
	CTX-M8 R	5′-AAC CCA CGA TGT GGG TAG C-3′		
***bla*CTX-M9**	CTX-M9 F	5′-CAA AGA GAG TGC AAC GGA TG-3′	205	[25,44]
	CTX-M9 R	5′-ATT GGA AAG CGT TCA TCA CC-3′		
***bla*CTX-M25**	CTX-M25 F	5′-GCA CGA TGA CAT TCG GG-3′	347	[25,44]
	CTX-M25 R	5′-AAC CCA CGA TGT GGG TAG C-3′		
***bla*CTX-M-15**	CTX3 FLF	5′-CGT CTC TTC CAG AAT AAG G-3′	924	[3]
	CTX3 FLR	5′-GTT TCC CCA TTC CGT TTC CGC-3′		

The amplified PCR products were subjected to electrophoresis at a 1 % agarose gel in 0.5X TBE buffer. The reading is done under UV light.

### Statistical analysis

Data extraction was performed using the epidemiological model of the Adagio Biorad antibiotic susceptibility testing system and the Laboratory Information System (LIS).

Statistical analysis was performed using Excel and SPSS version 25 software.

## Results

During the study period we collected 10 268 *Enterobacteriaceae* of which 1402 isolates were confirmed as producers of extended-spectrum β-lactamase with a prevalence of 13.65 %.

The predominance of ESBL-E infections was higher in men (57 %, i.e. *N*=799/1402) than in women (424 %, i.e. *N*=595/1402). The population’s mean age was 54 years with extremes between 0 and 105 years. Demographic and bacteriological characteristics of ESBL-E isolates are summarized in Tables3  4.

**Table 3.** Demographic and bacteriological data of ESBL-E isolates and CTX-M, TEM and SHV isolates

		All EBLSE	EBLSE CTX-M	EBLSE SHV	EBLSE TEM
**N (%**)		1 402/10 268(13.65 %)	105/110(95.45 %)	55/110(50 %)	62/110(56.36 %)
**Mean age**		54	60.30	57.37	59.02
**Sex**		♂: 57 %♀: 43 %	♂: 56 %♀: 44 %	♂: 63 %♀: 37 %	♂: 54 %♀: 46 %
**Germs**	*Klebsiella* (sp.) *pneumoniae*	*N*=592(42.2 %)	*N*=51(48.57 %)	*N*=46(83.63 %)	*N*=39(62.9 %)
	*Escherichia coli*	*N*=588(41.9 %)	*N*=45(42.85 %)	*N*=5(9.09 %)	*N*=19(30.64 %)
	*Enterobacter cloacae*	*N*=167(11.9 %)	*N*=8(7.61 %)	*N*=4(6.45 %)	*N*=4(6.45 %)
	*Proteus mirabilis*	*N*=21(1.5 %)	*N*=1(0.95 %)	*N*=0	*N*=0
	*Serratia* sp.	*N*=17(1.2 %)	*N*=0	*N*=0	*N*=0
	*Morganella morganii*	*N*=9(0.6 %)	*N*=0	*N*=0	*N*=0
	*Providencia* sp.	*N*=4(0.3 %)	*N*=0	*N*=0	*N*=0
	*Citrobacter* sp.	*N*=4(0.3 %)	*N*=0	*N*=0	*N*=0
**Sample**	Urine	*N*=858(61.2 %)	*N*=77(73.33 %)	*N*=40(72.73 %)	*N*=45(72.58 %)
	Pus	*N*=216(15.4 %)	*N*=13(12.38 %)	*N*=7(12.73 %)	*N*=8(12.9 %)
	Blood	*N*=92(6.6 %)	*N*=5(4.76 %)	*N*=4(7.27 %)	*N*=3(4.84 %)
	Pulmonary	*N*=91(6.5 %)	*N*=4(3.81 %)	*N*=1(1.82 %)	*N*=2(3.23 %)
	Swab	*N*=57(4.1 %)	*N*=1(0.95 %)	*N*=1(1.82 %)	*N*=1(1.61 %)
	Other	*N*=25(1.8 %)	*N*=0	*N*=0	*N*=0
	Catheter	*N*=22(1.6 %)	*N*=1(0.95 %)	*N*=1(1.82 %)	*N*=1(1.61 %)
	Puncture fluid	*N*=17(1.2 %)	*N*=1(0.95 %)	*N*=0	*N*=1(1.61 %)
	Faeces	*N*=10(0.7 %)	*N*=1(0.95 %)	*N*=0	*N*=0
	Materiel	*N*=8(0.6 %)	*N*=2(1.90 %)	*N*=1(1.82 %)	*N*=1(1.61 %)
	Biopsy	*N*=6(0.4 %)	*N*=0	*N*=0	*N*=0
**Origin**	Chirurgical	*N*=254(18.1 %)	*N*=24(22.86 %)	*N*=12(21.82 %)	*N*=10(16.13 %)
	Outpatients	*N*=235(16.8 %)	*N*=17(16.19 %)	*N*=6(10.91 %)	*N*=7(11.29 %)
	Medical	*N*=357(25.5 %)	*N*=23(21.90 %)	*N*=18(32.73 %)	*N*=14(22.58 %)
	Reanimation	*N*=147(10.5 %)	*N*=7(6.67 %)	*N*=4(7.27 %)	*N*=6(9.68 %)
	Emergency	*N*=334(23.8 %)	*N*=25(23.81 %)	*N*=12(21.28 %)	*N*=18(29.03 %)
	Not assigned	*N*=75(5.3 %)	*N*=9(8.57 %)	*N*=3(5.45 %)	*N*=7(11.29 %)

**Table 4.** Demographic and bacteriological data of ESBL-E CTX-M subgroup genes isolates

		EBLSE CTX-M1	EBLSE CTX-M2	EBLSE CTX-M8	EBLSE CTX-M9	EBLSE CTX-M25	EBLSE CTX-M15
**N (%**)		*N*=101/105(96.19 %)	*N*=1/105(0.95 %)	*N*=0/105	*N*=8/105(7.62 %)	*N*=0/105	*N*=82/105(78.10 %)
**Mean age**		60.91	33.00	–	56.00	–	61.89
**Sex**		♂: 54.45 %♀: 45.55 %	♂: 100 %♀: 0 %	–	♂: 62.5 %♀: 37.5 %	–	♂: 54.87 %♀: 45.13 %
**Germs**	*Klebsiella pneumoniae*	*N*=50(49.5 %)	*N*=0	–	*N*=2(25 %)	–	*N*=34(75 %)
	*Escherichia coli*	*N*=42(41.58 %)	*N*=1(100 %)	–	*N*=6(75 %)	–	*N*=41(75 %)
	*Enterobacter cloacae*	*N*=8(7.92 %)	*N*=0	–	*N*=0	–	*N*=6(75 %)
	*Proteus mirabilis*	*N*=1(0.99 %)	*N*=0	–	*N*=0	–	*N*=1(75 %)
	*Serratia sp*	*N*=0	*N*=0	–	*N*=0	–	*N*=0
	*Morganella morganii*	*N*=0	*N*=0	–	*N*=0	–	*N*=0
	*Providencia sp*	*N*=0	*N*=0	–	*N*=0	–	*N*=0
	*Citrobacter sp*	*N*=0	*N*=0	–	*N*=0	–	*N*=0
**Sample**	Urine	*N*=73(72.28 %)	*N*=1(100 %)	–	*N*=5(62.50 %)	–	*N*=59(71.95 %)
	Pus	*N*=13(12.87 %)	*N*=0	–	*N*=2(25 %)	–	*N*=10(12.2 %)
	Blood	*N*=5(4.95 %)	*N*=0	–	*N*=0	–	*N*=3(3.66 %)
	Pulmonary	*N*=4(3.96 %)	*N*=0	–	*N*=1(12.5 %)	–	*N*=4(4.88 %)
	Swab	*N*=1(0.99 %)	*N*=0	–	*N*=0	–	*N*=1(1.22 %)
	Catheter	*N*=1(0.99 %)	*N*=0	–	*N*=0	–	*N*=1(1.22 %)
	Puncture fluid	*N*=1(0.99 %)	*N*=0	–	*N*=0	–	*N*=1(1.22 %)
	Faeces	*N*=1(0.99 %)	*N*=0	–	*N*=0	–	*N*=1(1.22 %)
	Materiel	*N*=2(1.98 %)	*N*=0	–	*N*=0	–	*N*=2(2.44 %)
**Origin**	Chirurgical	*N*=24(23.76 %)	*N*=0	–	*N*=1(12.5 %)	–	*N*=20(24.39 %)
	Outpatients	*N*=17(16.83 %)	*N*=0	–	*N*=0	–	*N*=16(19.51 %)
	Medical	*N*=22(21.78 %)	*N*=1(100 %)	–	*N*=2(25 %)	–	*N*=16(19.51 %)
	Reanimation	*N*=7(6.93 %)	*N*=0	–	*N*=1(12.5 %)	–	*N*=3(3.66 %)
	Emergency	*N*=23(22.77 %)	*N*=0	–	*N*=2(25 %)	–	*N*=19(23.17 %)
	Not assigned	*N*=8(7.92 %)	*N*=0	–	*N*=2(25 %)	–	*N*=8(9.76 %)

**Fig. 1.**
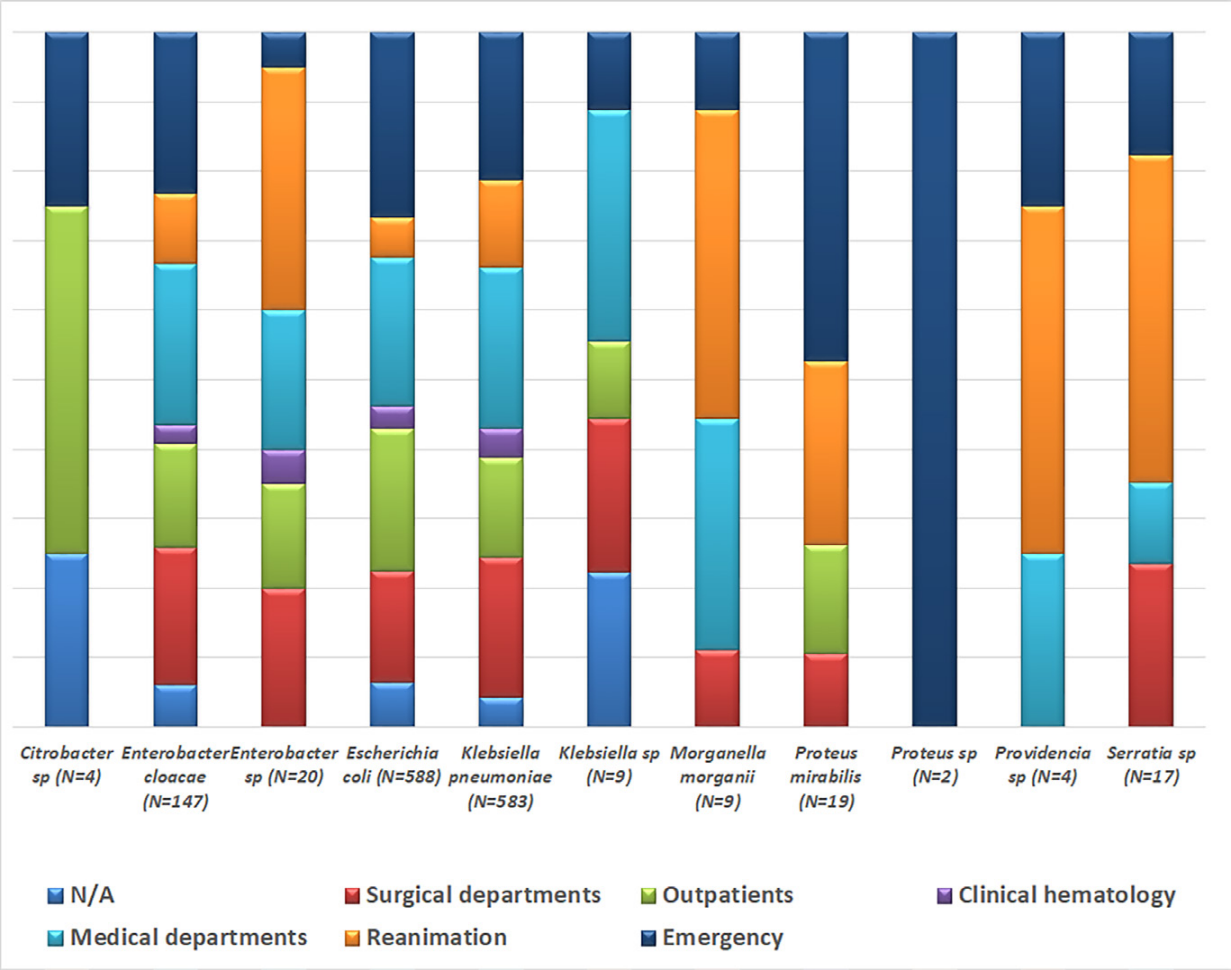
ESBL-E isolates distribution by department (*n*=1402). This figure shows that each department has its own bacterial epidemiology. *Klebsiella pneumoniae* is most prevalent in the medical, surgical and intensive care departments (hospital setting), while *Escherichia coli* is most isolated in outpatients and emergency departments (community setting).

**Fig. 2.**
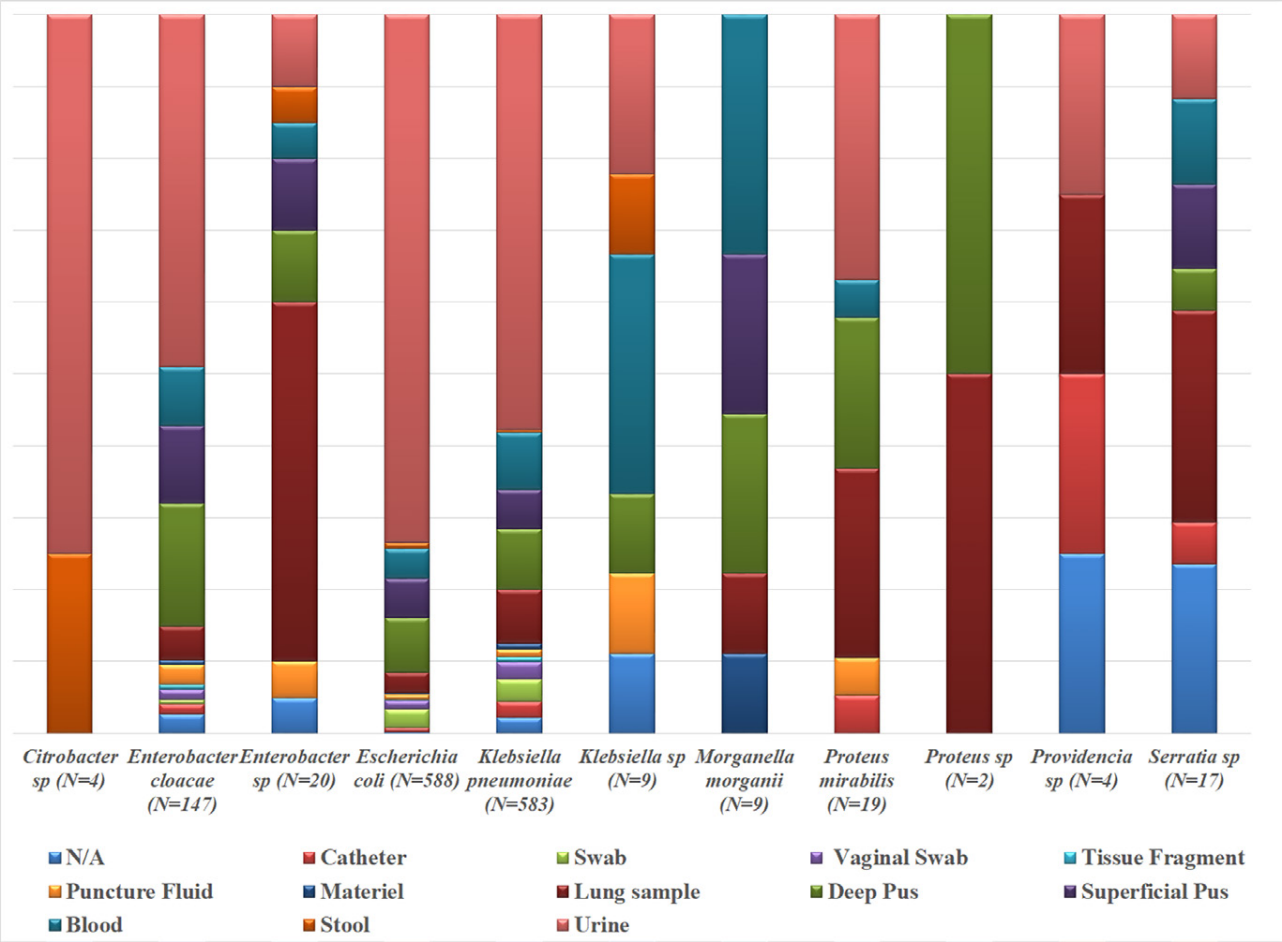
ESBL-E isolates distribution by sample (*n*=1402).This figure highlights the urinary tract as the most affected site, especially for *Escherichia coli*. Septicemia was marked by the presence of *Klebsiella pneumoniae* alongside nosocomial germs (*Morganella, Serratia*).

ESBL-E isolates were mostly represented by *Klebsiella pneumoniae* (41.5 %) and *Escherichia coli* (41.9 %), followed by *Enterobacter cloacae* (10.4 %) and *Proteus mirabilis* (1.3 %). The outpatients accounted for only 16.8 % of ESBL cases. The remaining majority was distributed in the different hospital departments; 25.5 % in medical department, 23.8 % in emergency department, 18.1 % in chirurgical department and 10.5 % in reanimation (Fig. 1). In the current work, urine samples were the most incriminated samples (61.2 %), pus samples were found in 15.4 % of cases, blood culture samples in 6.6%, lung samples in 6.5% and swab samples in 4.1 % as shown in Fig. 2.

All ESBL-E isolates exhibited resistance to ampicillin, first and third generation cephalosporins, while 88 % were resistant to amoxicillin-clavulanic acid, 24 % to ertapenem and 16 % to imipenem.

**Fig. 3.**
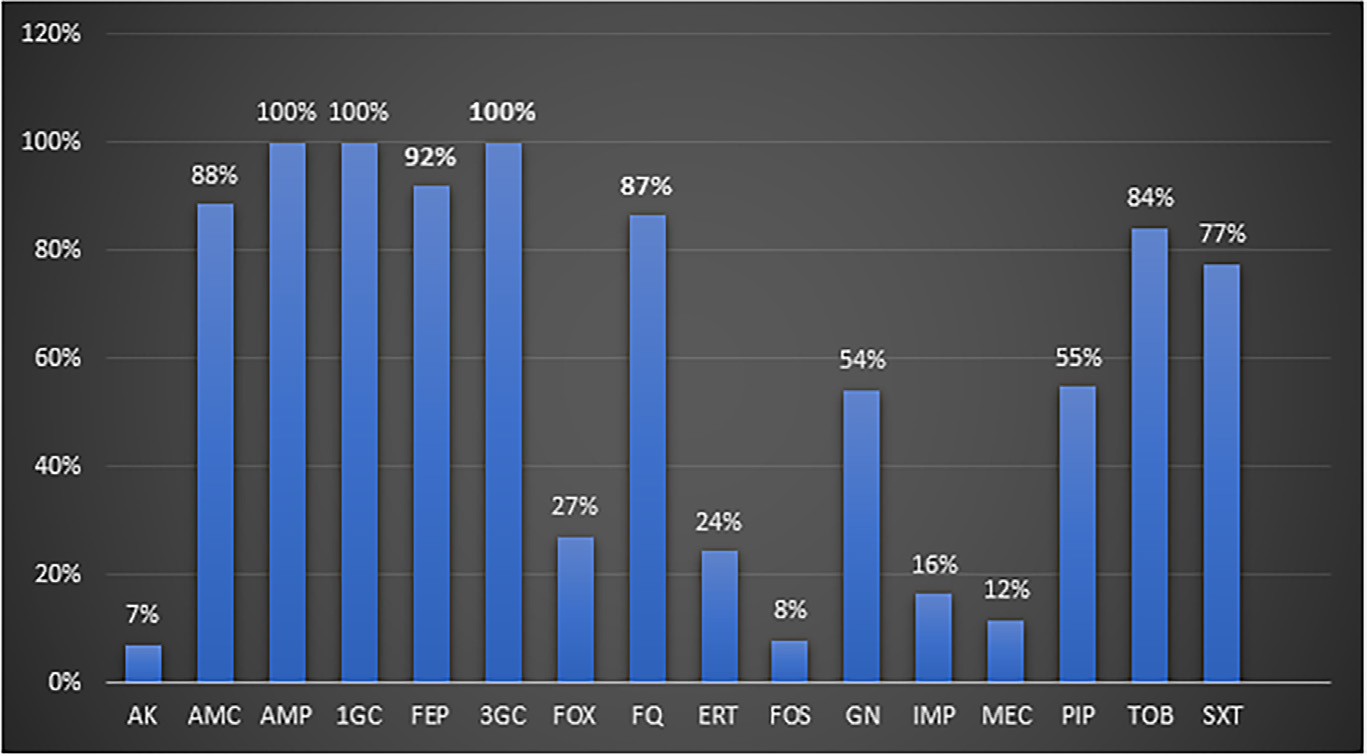
Resistance profile of ESBL-E isolates to antibiotics (*n*=1402). AK: amikacin, AMC: amoxicillin+clavulanic acid, AMP: ampicillin, 1GC: first generation cephalosporin, FEP: cefepim, 3GC: third generation cephalosporin, FOX: cefoxitin, FQ: fluoroqionolone, ERT: ertapenem, FOS: fosfomycin, GN: gentamicin, IMP: imipenem, MEC: mecillinam, PIP: piperacillin+tazobactam, TOB: tobramycin, SXT: trimethoprim / sulfamethoxazole.

The rates of resistance of ESBL-E isolates to fosfomycin and mecillinam were 8 and 16% respectively. In total, 87 % of the isolates were resistant to fluoroquinolones, 54 % to gentamycin while only 7 % were resistant to amikacin (Fig. 3).

Molecular typing of ESBL-encoding genes was performed only on a random representative sample. One hundred and ten isolates of phenotypically confirmed ESBL-producing *Enterobacteriaceae* were selected. CTX-M-type ESBL was the most common; 95.45% of the isolates possessed CTX-M gene, followed by TEM-type (56.36%) and SHV-type (50%).

We found 37.27 % of ESBL-E isolates (41/110) were positive for all three genes. In some cases, we observed the coexistence of two types of ESBL genes: CTX-M/TEM (18.18%), CTX-M/SHV (10.90%), SHV/TEM (0%). The production of a single gene concerned only 29% of the cases for CTX-M-type, 1.8% for SHV-type and only one isolate for TEM-type (Table 3).

The CTX-M-type ESBL-E positive isolates (105/110) were studied for the different subgroups CTX-M-1, -2, -8, -9, -25 and -15. The CTX-M-1 group and the CTX-M-9 group were the most common groups; 96.19 %(101/105) of ESBL isolates harboured CTX-M-1 gene and 7.62% (8/105) CTX-M-9 gene. The CTX-M-2 was positive for only one isolate and no CTX-M-8, CTX-M-25 groups were detected in the isolates. CTX-M-15 was detected in 78.10% (82/105) of cases. Most strains had more than two resistance genes (Table 4).

All the ESBL genes were male-dominant, were essentially isolated in urine samples and *Klebsiella pneumoniae* and *Escherichia coli* were the dominant species.

## Discussion

*Enterobacteriaceae* occupies an important place in bacterial infections, whether in hospitals or in the community. They constitute one of the most important families of bacteria, which are very heterogeneous in terms of pathogenesis and ecology.

This study was designed to investigate ESBL-E on a phenotypic and genotypic level. The prevalence of ESBL-producing *Enterobacteriaceae* was 13.65%, which is higher than those reported in other national studies [15,17]. A Chinese study found a higher rate (38%) than that shown by our study [9].

ESBL-E isolates were predominant in men (57%) with a sex ratio of M/F=1.32. This finding has been confirmed by several authors who reported that male gender and transfer from a long-stay hospital are two risk factors significantly associated with ESBL-E carriage [18].

ESBL isolates were dominated by *K. pneumoniae* and *E. coli* at equal percentages (42.2 % and 41.9%). Our rates are higher for *K. pneumoniae* than those reported in France (17%) [19] and those reported in hospitals in Southern Europe (25%) [20]. They are lower for *E. coli* compared to the results of studies conducted in France (≈67%) [21,22] and compared to the results of other studies (80 and 71 %) [16,23].

It was found that more than half of the samples came from urine samples. This finding was confirmed by the results of a study conducted in a university hospital in Paris by Lucet *et al*. Another study showed that 51% of ESBL-E were isolated from urine samples, the frequency of blood samples was high (15.3%) compared to our results [2].

The highest rate of our ESBL isolates was found in medical departments (25.5%), our rates in the different departments are close to the data of a French study which reported a frequency of 14% in intensive care units and 20% for patients consultants [21]. Indeed, patients hospitalized in intensive care units are at greater risk of contracting ESBL-E, given the length of hospitalization (which is generally long), the severity of the disease, the use of invasive devices (urinary catheters, catheters, ventilation, intubation, etc.) and the multiple antibiotic treatments, particularly with broad-spectrum cephalosporins [24].

The susceptibility profile of our ESBL isolates showed 100% resistance to first and third generation cephalosporins, 55% to piperacillin-tazobactam, 16% to imipenem, 24% to ertapenem, 87% to fluoroquinolones, 54% to gentamicin, 7% to amikacin, 12% to mecillinam, 8% to fosfomycin, and 77% to trimethoprim-sulfamethoxazole.

According to our study, resistance to carbapenems remains high compared to results of other studies [9,25]; the increase of ESBL-E isolates and the overuse of carbapenems in many countries has resulted in the emergence of resistance to these antibiotics, especially in *K. pneumoniae* [26].

Arpin *et al*. reported intermediate susceptibility or resistance to fluoroquinolones in 86% of cases, resistance to gentamicin in 29%, to amikacin in 51% and to trimethoprim-sulfamethoxazole in 86%. These figures remain comparable to our results with the exception of amikacin (antibiotic that remains active in 93% of our cases) [27]. Ben-Ami *et al*., in their review of the literature, had a fluoroquinolone resistance rate of over 70% [28].

This multidrug resistance could be explained by the fact that ESBL genes, usually carried by plasmids, are often associated with resistance genes to other antibiotics, especially aminoglycosides and fluoroquinolones [9]. A study conducted in Burkina Faso evaluated the frequency of qnr genes (quinolone resistance gene) in ESBL-E isolates and reported that the prevalence of the qnr-ESBL association was on average 27% [29]. Salah *et al*. confirmed in their study that of the 107 strains encoding qnr genes, 102, 96 and 52 carried CTX-M, TEM and SHV type ESBL respectively [30]. Therefore, cephalosporins and fluoroquinolones are not considered effective choices for the treatment of patients with ESBL-producing *Enterobacteriaceae* [9].

The combination of piperacillin and tazobactam remains active in 45% of cases, and therefore in these cases the use of carbapenems can be avoided. Several studies confirm the restoration of piperacillin sensitivity by tazobactam [31].

Fosfomycin has good antimicrobial activity against ESBL-E (sensitivity of 92%). This result was confirmed by a recent study that revealed the high efficacy of fosfomycin in the treatment of ESBL-E urinary tract infections, especially in community environment [32].

Several data have reported that the number of clinical isolates producing CTX-M has been steadily increasing in both hospital and community environment. TEM and SHV type ESBLs are continuously monitored in different hospitals worldwide, their numbers have been decreasing for many years and are increasingly replaced by CTX-M type ESBLs [33,34].

Limited data from nationwide studies have shown that ESBLs are common in Moroccan hospitals [15,35] and that the CTX-M, SHV and TEM genes represent the most frequently reported ESBL families in Morocco [36].

The results of our molecular study showed that the major mechanism of resistance of the ESBL-E isolates studied was the production of CTX-M ESBL (95% of the isolates carried the CTX-M gene). Isolates carrying more than two genes represented 66%, and 37% of cases were identified as positive for all three genes. The coexistence of more than one β-lactamase gene within the same isolate has also been reported in many other countries [27,37, 38]. CTX-M was found alone in 32 isolates (29% of cases). These results are in agreement with several data from the literature described in many European [12] and African [39] countries.

A study conducted in China reported that the CTX-M gene was identified in 126 isolates or a percentage of 96 %, the prevalence of TEM and SHV genes were less [9]. Al-Mayahie *et al*. reported that CTX-M ESBL were the most frequent (69.5 %), followed by SHV types (4.3 %) and no isolates had TEM ESBL [25].

We analysed all CTX-M-type positive strains (*N*=105) for subgroup genes by PCR assay. The results showed that almost all isolates (101/105) carried CTX-M-1 gene. Clearly, the CTX-M-1 type is the most common ESBL resistance gene, as indicated by most reports. The positivity rate of our CTX-M-1 isolates is higher than that reported abroad; Shu Xia *et al*. reported a positivity rate of 40.7% [40] and other studies pointed in the same direction with comparable rates [9,25]. Although the rate of our CTX-M-9 positive isolates is very low compared to the results of these studies.

At the same time, the main CTX-M gene identified was CTX-M-15; a variant of the CTX-M-1 subgroup, which is confirmed by most national [37,41, 42] and international researches [9,39]. This result suggests that CTX-M-15 is now common in Morocco and widely distributed among hospital infection patients, which is due in the first place to the wide use of β-lactam antibiotics and to hand-carrying dissemination.

ESBL enterobacterial infections leave only a slight limited choice in therapeutic management; several studies have discussed the alternatives and possibilities of antibiotic therapy in these infections. Many studies reported that β-lactam-β-lactamase inhibitor combination might be a reasonable option to spare carbapenems in the treatment of ESBL-producing *Enterobacteriaceae,* especially in less severe infections [9]. Several other therapeutic alternatives have been reported in the literature, namely temocillin, tigecycline and other combinations such as ceftolozane-tazobactam and ceftazidime-avibactam.

Our study had two major limitations: first, the non-availability of clinical information made it impossible to further analyse risk factors for ESBL-E infections, and second, the number of isolates studied was not large enough to predict epidemiological characteristics. Overall, the study was able to highlight the phenotypic (resistance to different antibiotics) and genotypic characterization of clinically isolated ESBL-E isolates. We found that CTX-M is still the main genotype of ESBL-E in Morocco and that the CTX-M-15 variant of the CTX-M-1 group is the most common type of resistance gene.

## Conclusion

Our study reports the occurrence of ESBL genes in pathogenic multidrug-resistant *Enterobacteriaceae* from hospitalized patients and outpatients in a Moroccan hospital. These isolates carried different β-lactamases and other resistance determinants. This is alarming as spread of these isolates will seriously limit options for clinical treatment in future. We hope that this phenotypic and molecular resistance data will help clinicians to better define the empiric treatment of infections caused by ESBL-E and to minimize the opportunity for their clonal diffusion.

## Abbreviations

CTX-M: cefotaxime-Munich
ESBL-E: extended-spectrum beta-lactamase-producing enterobacteriaceae
ESBLs: extended-spectrum beta-lactamases
1GC: 1st generation cephalosporins
3GC: 3rd generation cephalosporins
qnr genes: quinolone resistance gene
SHV: sulf-hydryl variable
